# Development and validation of a robust multigene signature as an aid to predict early relapse in stage I-III clear cell and papillary renal cell cancer

**DOI:** 10.7150/jca.38274

**Published:** 2020-01-01

**Authors:** Da-Long Cao, Wei-Xing Dai, Yong-Qiang Huang, Lei-Jun Yu, Jun-Long Wu, Guo-Hai Shi, Hai-Liang Zhang, Yao Zhu, Bo Dai, Ding-Wei Ye

**Affiliations:** 1 Department of Urology, Fudan University Shanghai Cancer Center, Shanghai 200032, China;; 2 Department of Oncology, Shanghai Medical College, Fudan University, Shanghai 200032, China;

**Keywords:** Renal cell carcinoma, Early relapse, Prognosis, Biomarker, mRNA signature

## Abstract

**Background and objectives:** Multi-gene signature can be used as prognostic indicator in many types of cancer, but the association with early-relapse in patients with stage I-III clear cell and papillary renal cell cancer (RCC) is unknown. We aim to establish a mRNAs signature for improving prediction of early-relapse in patients with stage I-III clear cell and papillary RCC.

**Methods:** The data of 610 patients with stage I-III RCC from The Cancer Genome Atlas (TCGA) and 270 patients from Fudan University Shanghai Cancer Center (FUSCC) were extracted. Propensity score matching analysis, linear models for microarray data VOOM method, least absolute shrinkage and selection operation Cox regression modeling analysis was conducted in turn for selecting multi-mRNA signature. Survival differences were assessed by Kaplan-Meier estimate and compared using log-rank test. Multivariable Cox regression and time-dependent receiver operating characteristic curves were used to evaluate the association of mRNAs signature with relapse-free survival (RFS).

**Results:** Seventeen mRNAs were identified to constitute the early-relapse signature. Among patients with stage I-III RCC, those with high-risk score calculated from 17 mRNAs signature showed shorter RFS than those with low-risk score, both in TCGA discovery and internal validation sets, and in FUSCC discovery and internal validation sets (all p < 0.05). In multivariable Cox regression analysis, the 17 mRNAs signature remained an independent prognostic factor both in TCGA discovery (HR 2.43, 95%CI 1.98-2.96) and internal validation sets (HR 1.66, 95%CI 1.19-2.30), and FUSCC discovery (HR 1.28, 95%CI 1.13-1.43) and internal validation sets (HR 1.65, 95%CI 1.11-2.48). Additionally, the 17 mRNAs signature achieved a higher accuracy for RFS estimation beyond clinical indicator.

**Conclusion:** The 17 mRNAs signature could classify stage I-III RCC patients into low- or high-risk of early-relapse, and will help to guide interventions to optimize survival outcomes.

## Introduction

Renal cell cancer (RCC) is one of the worldwide common carcinomas, with approximately 403,262 new cases and 175,098 deaths expected in 2018^1^. The overall prognosis of RCC could be improved from the implement of curative resection, which is the benchmark for the treatment of RCC. However, approximately 20-30% of all patients treated with adequate surgical excision subsequently experience recurrence or metastases during follow-up^2^, ^3^. The relapse of RCC is time-related, of which the greatest recurrence risk is in the first 5 years after surgery and only 10% of recurrences occur after 5 years from nephrectomy^4^-^6^. Early relapse in RCC is related to more symptoms at presentation, larger tumor size, and aggressive histology and pathological stage^7^, ^8^, and naturally patients developed early relapse consistently tended to have poorer over survival than those with late recurrence 5 years after nephrectomy^8^. Consequently, more valuable predictive factors are urgently needed to distinguish patients with early post-operative relapse.

Current clinical tools to stratify patients with RCC are limited to a set of clinical and pathologic variables (such as the TNM staging system), which are unable to reflect the biological heterogeneity of cancer^9^. As reason described above, prognosis even varies significantly in RCC patients with comparable clinicopathological characteristics and same tumor TNM stage. Despite researchers are exploring extensively the potential indicator or biomarker for predicting early relapse in RCC patients^10^-^12^, none of gene-based prognostic classifiers for predicting early relapse of RCC have been established. Although studies in clear cell RCC demonstrated that gene signature has better ability to both reflect heterogeneity of cancer and then accurately predict cancer prognosis 13-15. These studies were limited to overall survival (OS)-related genes in clear cell RCC, and few precious gene profiling has been applied to detect the early relapse-associated multigene signature in both clear cell and papillary RCC. Because OS stands for all-cause mortality, there is a critical need for improved prognostic discrimination in RCC patients given the increasing awareness that some patients may be managed with active surveillance, while others with high-risk of early-relapse might benefit from adjuvant therapy following surgery. Therefore, exploring a novel gene signature to identify early relapse in clear cell and papillary RCC patients might be of concrete predictive value.

In this study, we adopted previously published gene expression data from The Cancer Genome Atlas (TCGA) project and conducted mRNA profiling on large cohorts of RCC patients. Using the sample splitting method and Cox regression analysis, a prognostic 17-mRNA signature was identified from the discovery set and validated in the internal validation series and the external cohorts, and could provide additional prognostic information beyond standard clinical parameters, offering a new approach for risk stratification. This 17-mRNA signature could help distinguish the subset of stage I-III clear cell and papillary RCC patients at high risk of early relapse, who should be managed with extensive postoperative treatment and surveillance.

## Materials and methods

### Patient cohorts

For the discovery set and internal validation set, a total of 610 stage I-III RCC patients were obtained from TCGA database with available RNA sequencing data and clinical annotation. For the external validation set, pathologically diagnosed and RNA*later* Stabilization Solution-stored tissue samples of 270 patients with stage I-III RCC were obtained from Fudan University Shanghai Cancer Center (FUSCC). The clinical characteristics of patients from FUSCC dataset were summarized in **Table 1**. This study was approved by the Ethical Committee of FUSCC, and written informed consent was obtained from all patients.

### Developing early relapse associated gene signature

**Figure 1:**
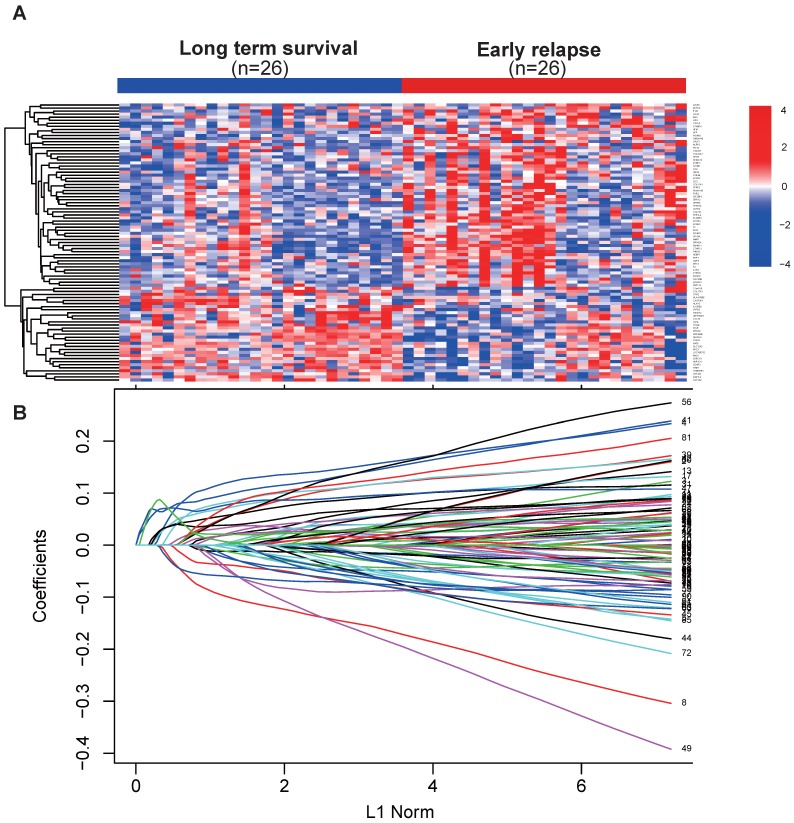
(A) Heat map showed 91 differentially expressed mRNA in renal cell cancer between early relapse and long-term survival group in discovery set from TCGA dataset. (B) LASSO coefficient profiles of the 91 early relapse-associated mRNA. A vertical line is drawn at the value chosen by 10-fold cross-validation.

Early relapse was defined as the locoregional recurrence or distant metastasis within 2 years after surgery. Samples in the discovery set from TCGA were selected and divided into early relapse group and long-term survival group (no relapse after a minimum of 5 years follow-up). Propensity score (PS) matching analysis was performed between the two groups to adjust for stage and histological type, which were the most significant clinical factors associated with early relapse. After PS matching, 26 paired patients were finally selected to detect the changes of global gene expression profile between early relapse and long-term survival groups **(Table 2)**. Next, using the linear models for microarray data (LIMMA) VOOM method for identification of differentially expressed genes (DEGs) with the threshold set as *P* < 0.05 and fold change ≥ 2.5, we found that 91 genes were differentially expressed between early relapse and long-term survival samples **(Fig. 1A)**. Using LASSO Cox regression model^16^, the coefficient profiles of the 91 genes were obtained and shown in **Figure 1B** and then 17 mRNAs were picked out to construct the 17 mRNAs-based signature. Finally, we derived a formula to calculate the risk score for predicting the early relapse based on the individual expression of the 17 mRNAs weighted by the regression coefficient in the discovery set as follows: 17 mRNA signature risk score = (0.183 × expression level of AFP) + (- 0.216 × expression level of ATP6V0D2) + (0.112 × expression level of COL22A1) + (0.064 × expression level of EN2) + (0.047 × expression level of EYA1) + (- 0.026 × expression level of HOXA13) + (0.102 × expression level of IGF2BP3) + (0.142 × expression level of IGSF9) + (- 0.051 × expression level of ITGAD) + (0.115 × expression level of KCNG1) + (0.135 × expression level of MT1X) + (0.107 × expression level of PGAM2) + (- 0.062 × expression level of RYR2) + (- 0.082 × expression level of SLC22A2) + (0.048 × expression level of STRA6) + (0.024 × expression level of STXBP6) + (- 0.022 × expression level of ZIC2).

To assess the 17 mRNA signature using quantitative reverse transcription polymerase chain reaction (RT-PCR) analysis, we recalculated the regression coefficients of the 17 mRNAs based on univariable Cox regression analysis from quantitative RT-PCR expression data in FUSCC population. The primers for RT-PCR were summarized in **Supplemental Table 1**. A new risk score formula was derived with the same method and mRNAs used in the discovery set as follows: 17 mRNA signature risk score = (0.049 × expression level of AFP) + (- 0.816 × expression level of ATP6V0D2) + (0.213 × expression level of COL22A1) + (0.464 × expression level of EN2) + (0.660 × expression level of EYA1) + (- 0.424 × expression level of HOXA13) + (0.102 × expression level of IGF2BP3) + (0.107 × expression level of IGSF9) + (- 0.024 × expression level of ITGAD) + (0.113 × expression level of KCNG1) + (0.188 × expression level of MT1X) + (0.318 × expression level of PGAM2) + (- 0.287 × expression level of RYR2) + (- 0.283 × expression level of SLC22A2) + (0.358 × expression level of STRA6) + (0.207 × expression level of STXBP6) + (- 0.319 × expression level of ZIC2).

In the two formulas, same mRNA has consistent risk prediction directions, suggesting that the classifier could apply to both RNA-seq and RT-PCR data.

### Statistical analysis

With risk score formula, patients from different sets were divided into high-risk and low-risk groups using the median risk score as the cutoff point. The difference between two groups was compared using x^2^ test or Fisher's exact test for categorical variables and t test for numerical variables. Survival differences between the low-risk and high-risk groups in each set were assessed by the Kaplan-Meier estimate and compared using the log rank test. Multivariate Cox regression analysis and data stratification analysis were performed to test the independent prognostic role of risk score in predicting RFS. Time-dependent receiver-operating characteristic (ROC) analysis was used to investigate the predictive accuracy of each feature and multi-gene signature. All statistical analyses were performed with use of R (version 2.15.0, www.r-project.org). All statistical tests were two-sided, and *P* values < 0.05 were considered statistically significant.

## Results

Among 610 patients from TCGA dataset, 428 and 182 patients were randomly assigned into the discovery set and internal validation set, respectively. In FUSCC population, 180 and 90 patients were respectively assigned into the training and validation sets.

**Figure 2:**
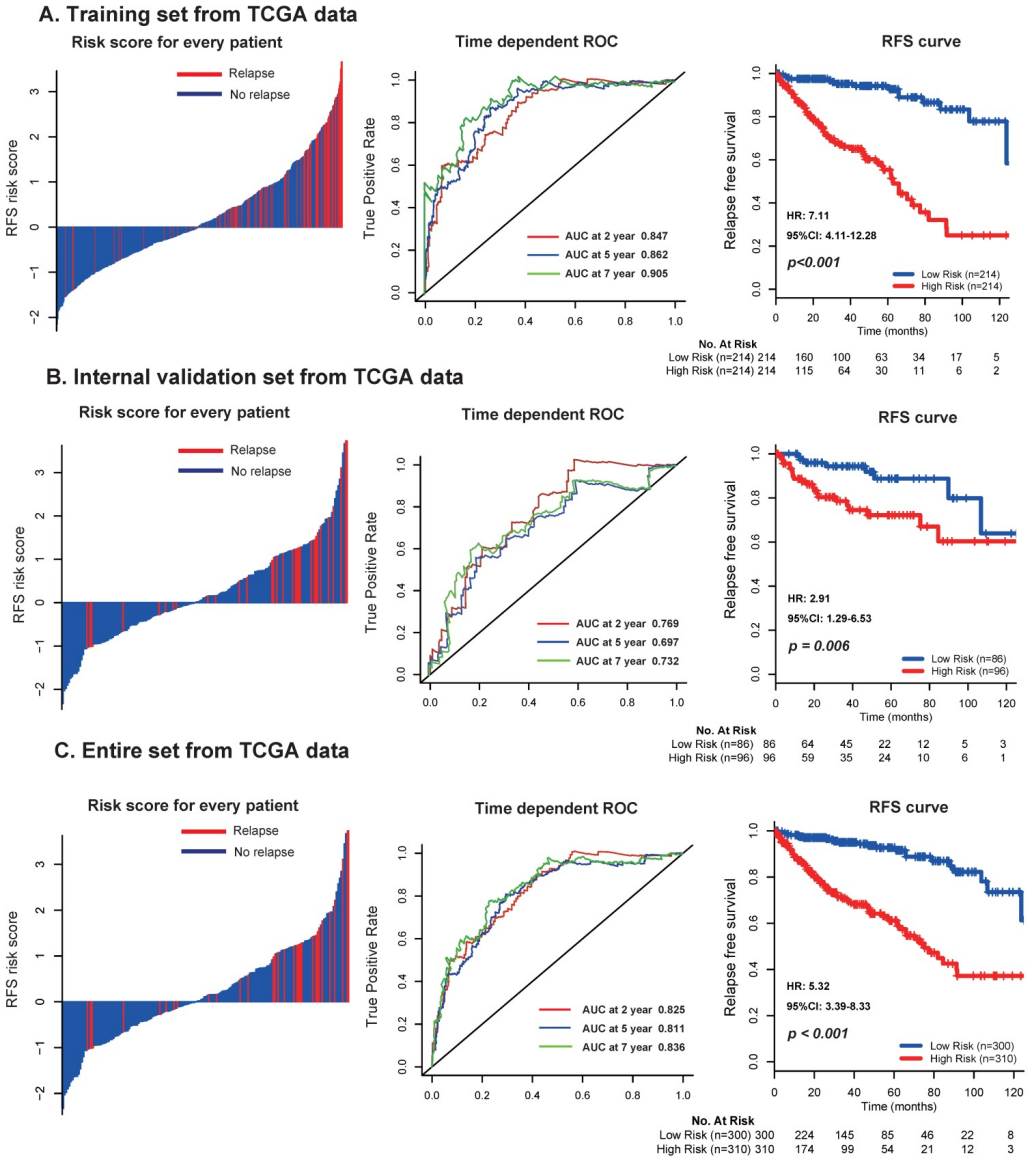
Distribution of risk score, time-dependent ROC curves at 2, 5, and 7 years and Kaplan-Meier survival analysis between patients with low and high risks of relapse in discovery set (A), internal validation set (B), and entire dataset (C) from TCGA data. TCGA: The Cancer Genome Atlas (TCGA) dataset.

In the discovery set from TCGA dataset, patients were divided into low risk group (n=214) and high risk group (n=214) using the median risk score (1.611) as cutoff point. As shown in **the left panel of Figure 2A**, the distribution of risk scores and survival status suggested that patients with higher risk scores tended to have earlier relapse than those with lower risk scores. Using time-dependent ROC analysis, the prognostic accuracy of the 17 mRNA signature for RFS at 2, 5, 7 years were respectively calculated and confirmed (AUC = 0.847, 0.862 and 0.905, respectively;** Fig. 2A, middle panel**). Patients with high risk scores had worse RFS than those with low risk scores (2-year RFS: 76% vs 97.5%, 5-year RFS: 55.3% vs 94.3%, 7-year RFS: 32.1% vs 86.6%, *p* < 0.001; **Fig. 2A, right panel**). We furtherly applied the same analyses, same formula and cutoff point in the TCGA internal validation set and entire set, and obtained consistent results **(Fig. 2B-C)**.

**Figure 3:**
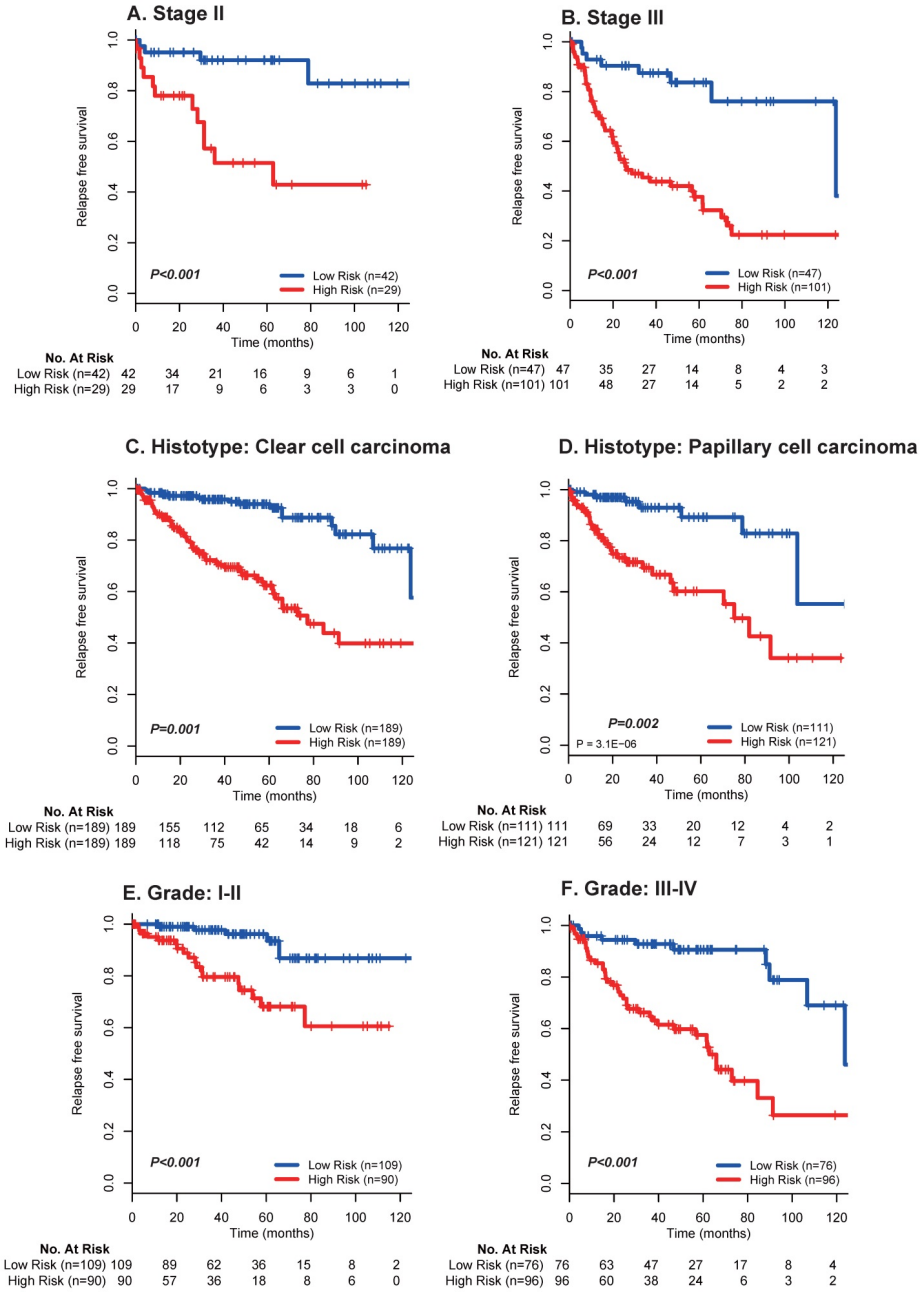
Kaplan-Meier survival analysis for the entire set with stage I-III renal cell cancer (N = 610) from TCGA data based on the 17-mRNA-based signature stratified by stage II (A), stage III (B), clear cell carcinoma (C), papillary cell carcinoma (D), Grade I-II (E) and Grade III-IV (F). TCGA: The Cancer Genome Atlas (TCGA) dataset.

Moreover, multivariate analyses showed that the 17 mRNA signature remained a powerfully and independently prognostic factor for RFS in the discovery set [hazard ratio (HR): 2.43, 95% confidence interval (CI): 1.98-2.96, *p* < 0.001] and internal validation set (HR: 1.66, 95%CI: 1.19-2.30, *p* = 0.002) from TCGA dataset after adjustment by clinicopathological features **(Table 3)**. Importantly, stratified analyses also suggested that the 17 mRNA classifier was still a clinically and statistically significant prognostic indicator for RFS in subset of patients with stage II **(*p* < 0.001; Fig. 3A)** and stage III **(*p* < 0.001; Fig. 3A)**, patients with clear cell carcinoma **(*p* = 0.001; Fig. 3B)** and papillary carcinoma **(*p* = 0.002; Fig. 3B)**, patients with grade I-II **(*p* < 0.001; Fig. 3C)** and grade III-IV **(*p* < 0.001; Fig. 3C)**. These evidences demonstrated that our 17 mRNA signature could screened out high risk patients from those with better clinic-prognostic factors (e.g. early stage, clear cell cancer and low grade) and low risk patients from those with poor clinicopathological variables (e.g. advanced stage, papillary cell cancer and high grade), and ultimately optimize the risk prediction of patients' early relapse and survival in clinical practice.

**Figure 4:**
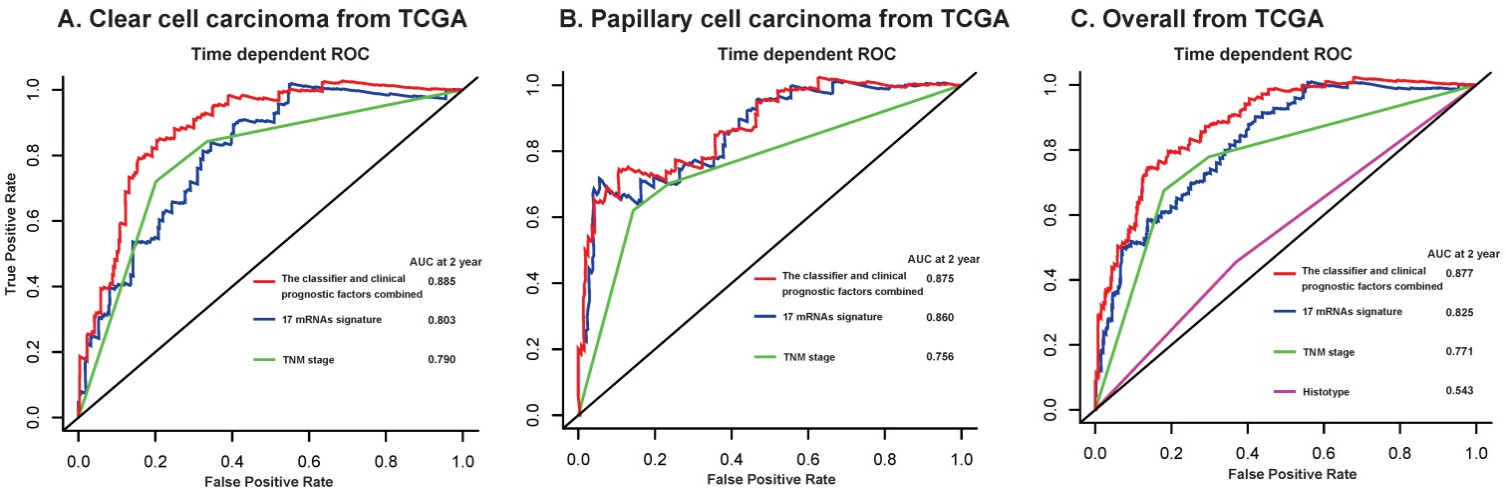
Time-dependent ROC curves at 2 year compare the prognostic accuracy of the 17-mRNA signature in predicting early relapse with TNM staging system and histological type in stage I-III patients with clear cell carcinoma (A) and papillary cell carcinoma (B), and in the entire patients (C) from TCGA data. TCGA: The Cancer Genome Atlas (TCGA) dataset.

Furthermore, we compared our 17-mRNA classifier with the existing clinic-prognostic factors using time-dependent ROC analysis. The 17-mRNA classifier showed superiority in predicting RFS compared with TNM stage **(AUC at 2 years: 0.803 vs 0.790 in clear cell cancer; 0.860 vs 0.756 in papillary cell cancer; 0.825 vs 0.771 in entire patients; Fig. 4)**, and histological type **(AUC at 2 years: 0.825 vs 0.543 in entire patients; Fig. 4)**. Importantly, the combination of 17-mRNA signature with the clinic-prognostic factors improved the predictive accuracy of RFS at 2 years **(AUC at 2 years: 0.885 in clear cell cancer, 0.875 in papillary cell cancer, 0.877 in entire patients; Fig. 4)**, suggesting that the 17-mRNA classifier could add complementary value to clinically prognostic indicators.

**Figure 5:**
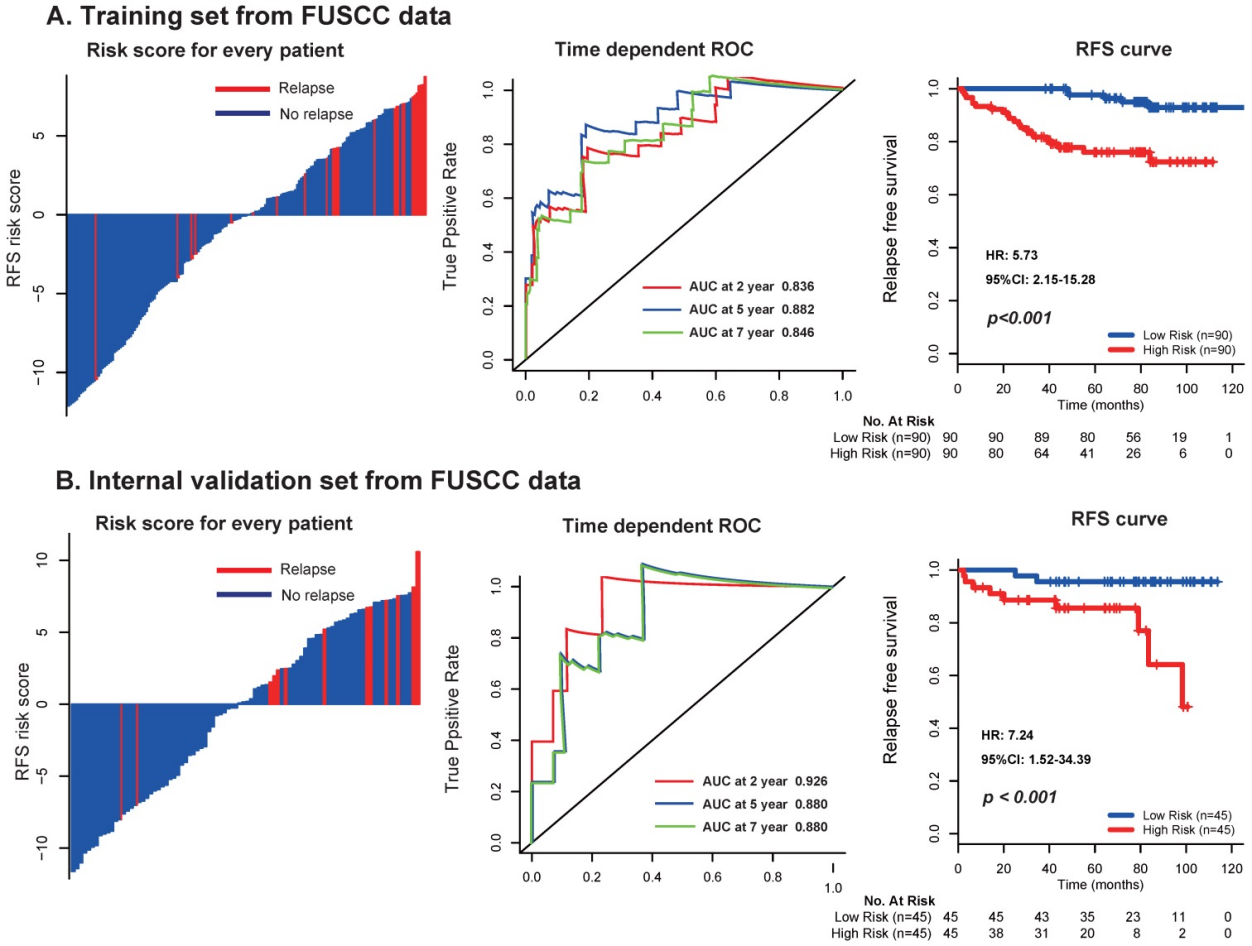
Distribution of risk score, time-dependent ROC curves at 2, 5, and 7 years and Kaplan-Meier survival analysis between patients with low and high risks of relapse in discovery set (A) and internal validation set (B) from FUSCC data. FUSCC: Fudan University Shanghai Cancer Center.

**Figure 6:**
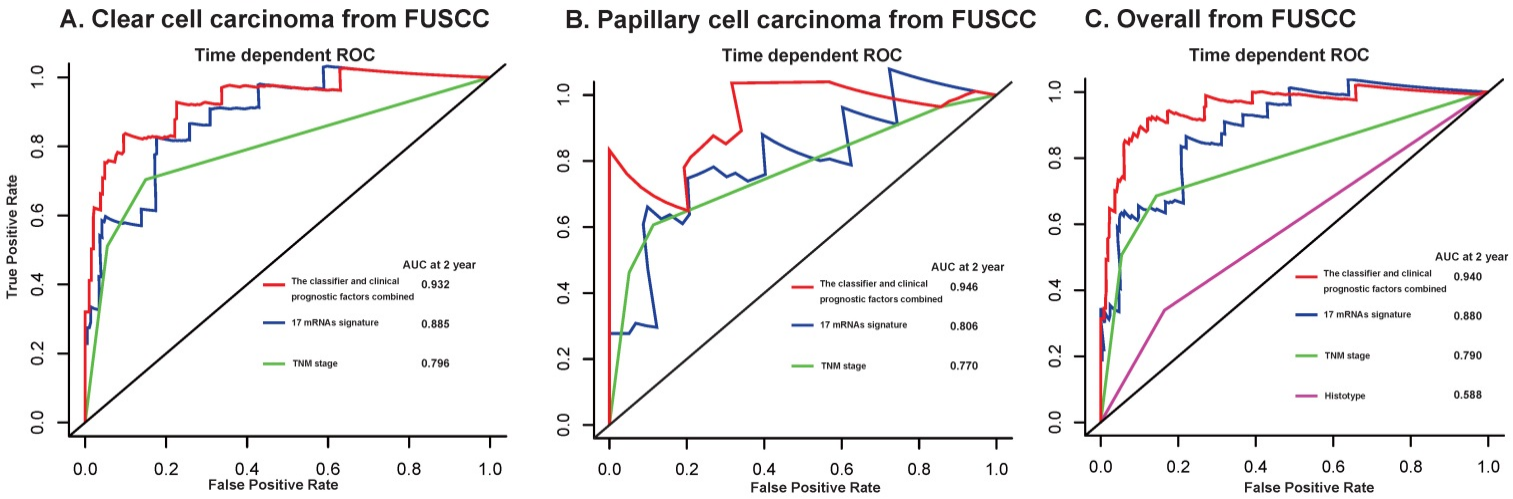
Time-dependent ROC curves at 2 year compare the prognostic accuracy of the 17-mRNA signature in predicting early relapse with TNM staging system and histological type in stage I-III patients with clear cell carcinoma (A) and papillary cell carcinoma (B), and in the entire patients (C) from FUSCC data. FUSCC: Fudan University Shanghai Cancer Center.

To further assess the robustness of the signature, we determined the 17-mRNA classifier by RT-PCR analysis in the FUSCC population. Due to the difference of RT-PCR quantification and RNA-seq technique, a new formula for the RT-PCR data were development in the training set and validated in the internal validation set from FUSCC population by the same method used in the discovery set from TCGA dataset. With this risk score formula, patients were stratified into high- or low-risk groups with a median risk score of 0.354 as cutoff point. Both in the training set and the internal validation set, patients with high-risk scores generally tended to have earlier relapse and worse RFS than those with low-risk scores **(Fig. 5)**, and the prognostic accuracy of the 17 mRNA signature for RFS were furtherly confirmed by time-dependent ROC analysis **(Fig. 5)**. In the univariable and multivariable Cox regression analyses, the 17 mRNAs signature was still an independent prognostic factor for RFS in FUSCC cohort **(Table 4)**. Similarly, we found that the 17-mRNA classifier still showed superiority in predicting RFS compared with the existing clinic-prognostic factors both in patients with clear cell cancer and papillary cell cancer, and in entire patients **(Fig. 6)**.

Finally, gene set enrichment analysis (GSEA) was performed to identify the 17-mRNA signature associated biological function and signal pathway. The risk score was accompanied with exceptional regulation of several important cancer-related networks, namely P53 signaling pathway, cell cycle, citrate cycle (TCA cycle), fatty acid metabolism, PPAR signaling pathway. The biological function of these 17 mRNAs in RCC should be investigated in further experimental studies.

## Discussion

Postoperative relapse in localized RCC patients still occurs even after complete surgical resection and is closely associated with survival outcomes^17^. However, early and late relapse after surgery cannot be distinguished by TNM staging system which mainly depends on anatomical information instead of biological characteristics. The large variation in the relapse and prognosis of localized RCC patients with same clinicopathological features is attributed to the biological heterogeneity of cancer^10^-^12^. RCC patients with early relapse suffer from significantly poor OS rates comparing to those with late relapse^18^. Novel prognostic biomarkers for the detection of early postoperative relapse would make up for the deficiency of TNM staging system, and thereby assisting physicians in formulating more efficient therapeutic strategies at an earlier stage of patients' treatment^18^-^20^. In this study, we developed and validated a novel gene signature based on 17 mRNAs to improve the prediction of early relapse and relapse-free survival (RFS) after surgery for stage I-III RCC patients. This 17 mRNA signature was independent of known clinical predictors, suggesting that this established predictor adds additional prognostic information beyond currently available tumor characteristics.

Previous studies have tried to identify biomarkers for detection of early relapse in RCC patients. In 2012, Slaby et al 12found that the expression levels of miR-145 and miR-126 were significantly associated with early relapse and survival in RCC patients. Additionally, it is also suggested in 2017 that CD8^+^PD-1^+^Tim-3^+^Lag-3^+^ tumor-infiltrating lymphocytes, ICOS^+^ tumor-infiltrating Treg cells may be as significant factors for postoperative early relapse in localized RCC patients^10^. Moreover, the recurrence score based on 16 genes was found to be a more accurate and individual predictor of clinical outcome in stage I-III clear cell RCC patients. However, these works have not focused on the postoperative early relapse. Little is known about mRNA expression penal and its involvement in the prediction of early relapse in stage I-III RCC patients using high-throughput expression profile datasets.

Importantly, we detected that RFS in patients with high risk of early relapse calculated using 17 mRNA signature were significantly worse than those with low risk of early relapse in the TCGA discovery set. It was also validated both in the internal validation series of TCGA dataset and in the independent set from FUSCC population, indicating that the good reproducibility of this 17 mRNA signature in RCC patients. Meanwhile, patients with same TNM stage (stage II or stage III), same pathological type (clear cell or papillary cell cancer), or same grade (grade I-II or grade III-IV) could be stratified into different risk groups based on the 17 mRNA classifier, which could lead to more personalized treatment for RCC patients to improve clinical outcomes. This findings implied that the 17 mRNA signature could be used to optimize the current risk stratification (e.g. TNM stage), and patients with high risk of early relapse might be benefit from more aggressive treatments^20^.

Several other groups have only focused on classifiers related with recurrence and death in clear cell RCC. Brooks and colleagues^13^ found that a classifier, ClearCode34, demonstrated improved prognostic performance over baseline nomograms and a c-indices of 0.65-0.70. Another classifier, a 16-gene assay, was found to be independently associated with cancer recurrence with c-index of 0.81^14^. In contrast to these signature development studies, the current study mainly focused on the early relapse both in clear cell and papillary RCC. And we found that our 17 mRNA signature demonstrated a AUC of 0.825 in TCGA and 0.880 in FUSCC cohorts in predicting early relapse at 2 years after complete resection of RCC. Even integrating this 17 mRNA signature with clinic-prognostic factors has the best prognostic accuracy (AUC = 0.877 and 0.940 in TCGA and FUSCC datasets, respectively) in our study. Therefore, this 17 mRNA signature developed in our study, which could help distinguish RCC patients with high risk of early relapse and then guide personalized management, is credible to be applied to clinic.

Previous studies have demonstrated that the most aggressive RCC are characterized by reduced angiogenic dependence^21^, ^22^, deteriorative immune and inflammatory responses^23^, ^24^, deregulated glycolysis and tricarboxylic acid cycle^25^, and increased cell proliferation^26^. Similarly, genes identified in our 17 mRNA signature are involved in biological pathways known to be important to the biology of RCC, namely P53 signaling pathway, cell cycle, citrate cycle (TCA cycle), fatty acid metabolism, PPAR signaling pathway. Some of the known biomarkers (e.g. von hippel lindau, hypoxia-inducible factor, MET, and PDL1) in RCC were not significantly correlated with early relapse at the RNA level, because it is known that changes in DNA are not always reflected by differential RNA expression and that mutations related with tumor are not necessarily associated with clinical outcome^27^. Thus, it is a plausible explanation for the association of the 17 mRNA signature with early relapse of RCC patients.

The heterogeneity of cancer has recently been discussed as a potential challenge in the use of genomic-based prognostic and predictive markers. Previous sequencing data have detected that the degree of tumor heterogeneity in RCC is substantial^28^. Genetic profiling of nine areas in the primary tumor and three metastatic sites in one individual patient indicated that 23% of identified mutations were restricted to that patient and not prevalent in RCC tumors in general. However, this result implies that approximately 77% of somatic mutations were common to other RCC. The consistent performance of early relapse score calculated by the selected genes across large cohorts and relevant subgroups supported low intra-tumor variability in the 17 assessed genes. Similar to ubiquitous somatic mutations in the study by Gerlinger et al^28^, the selected genes may represent early genetic changes in tumor development.

Limitations of our study warrant further discussion. Firstly, our research was based on the data from publicly available datasets, additional sets of independent samples from clinical trials are needed to prospectively confirm our findings. Meanwhile, several other important clinicopathological characteristics (e.g. SSIGN score) were not available in sets of the current study, and further analysis stratified by these features are necessary in the future research. Moreover, the mechanism behind the identified 17 mRNA signature on the early relapse in RCC is unclear, and further studies of these genes may provide more clues that leads to a better understanding of the early relapse and progression in RCC patients. We acknowledge that prospective, large-scale, multicentre studies are necessary to confirm our results before this 17 mRNA signature can be really applied in the clinic.

To the best of our knowledge, we firstly developed a robust mRNA signature that can effectively classify stage I-III RCC patients into groups with low and high risks of postoperative early relapse. Therefore, the 17 mRNA classifier, which can be combined with clinicopathological parameters, allows for risk assessment of early relapse in RCC patients and guides future clinical planning regarding patients' treatment and surveillance.

## Supplementary Material

Supplementary tables.

## Figures and Tables

**Table 1:** Basic features of renal cell cancer patients in FUSCC database.

Variables	Training set		Validation set		*P*
	N	%	N	%	
**Age**					0.221
<60	110	61.1	48	53.3	
≥60	70	38.9	42	46.7	
**Sex**					0.503
Female	53	29.4	23	25.6	
Male	127	70.6	67	74.4	
**Grade**					0.438
I-II	89	49.4	49	54.4	
III-IV	91	50.6	41	45.6	
**Stage**					0.351
I	144	80	69	76.7	
II	20	11.1	8	8.9	
III	16	8.9	13	14.4	
**Histological type**					0.438
ccRCC	149	82.8	71	78.9	
pRCC	31	17.2	19	21.1	

FUSCC: Fudan University Shanghai Cancer Center; ccRCC: clear cell renal cell cancer; pRCC: papillary renal cell cancer.

**Table 2:** Clinical-pathological features of patients in early relapse and long-term survival groups before and after propensity score matching in TCGA database.

Variable	Training Set					
	Before matching			After matching		
	early relapse	long-term survival	p	early relapse	long-term survival	p
**Age**(mean, IQR)	62.9	58.1	0.049	65.3	60.27	0.810
	(54.0-74.0)	(49.0-66.0)		(56.3-75.0)	(53.0-67.0)	
**Gender**			0.570			0.064
female	17	31		11	8	
male	30	44		15	18	
**Stage**			**<0.001**			**1**
I	8	49		8	8	
II	6	13		5	5	
III	33	13		13	13	
**Histological type**			**<0.001**			**1**
Clear cell carcinoma	24	60		14	14	
Papillary cell carcinoma	23	15		12	12	
**Total**	47	75		26	26	

TCGA: The Cancer Genome Atlas (TCGA) project.

**Table 3:** Univariable and multivariable Cox regression analysis in renal cell cancer from TCGA database.

Variables	Univariate Analysis		Multivariate Analysis	
	HR(95%CI)	p	HR(95%CI)	p
**Training set(N=428)**				
Age	1.02(1.00-1.04)	0.025	1.01(0.99-1.03)	0.132
17 gene risk score	2.68(2.22-3.25)	<0.001	2.43(1.98-2.96)	<0.001
Gender				0.857
female	1	0.223	1	
male	1.34(0.84-2.14)		1.05(0.65-1.69)	
Stage		<0.001		<0.001
I	1		1	
II	2.78(1.45-5.31)		2.45(1.25-4.82)	
III	5.53(3.45-8.86)		3.45(2.11-5.65)	
Histological type		0.184		
ccRCC	1		1	0.019
pRCC	1.34(0.87-2.07)		1.75(1.09-2.80)	

**Internal validation set(N=182)**				
Age	0.99(0.97-1.03)	0.853	1.01(0.98-1.03)	0.648
17 gene risk score	1.82(1.34-2.47)	<0.001	1.66(1.19-2.30)	0.002
Gender		0.538		0.804
female	1		1	
male	0.78(0.37-1.68)		0.91(0.42-1.96)	
Stage		0.002		0.031
I	1		1	
II	0.63(0.14-2.83)		0.90(0.19-4.09)	
III	3.30(1.57-6.94)		2.73(1.25-5.97)	
Histological type		0.890		0.929
ccRCC	1		1	
pRCC	1.05(0.49-2.25)		1.04(0.47-2.30)	

TCGA: The Cancer Genome Atlas (TCGA) project; ccRCC: clear cell renal cell cancer; pRCC: papillary renal cell cancer.

**Table 4:** Univariable and multivariable Cox regression analysis in renal cell cancer from FUSCC database.

Variables	Univariate Analysis		Multivariate Analysis	
	HR(95%CI)	p	HR(95%CI)	p
**Training set(N=180)**				
Age		0.530		0.620
<60	1		1	
≥60	1.28(0.59-2.77)		1.23(0.54-2.81)	
17 gene risk score	1.27(1.11-1.43)	<0.001	1.28(1.13-1.43)	<0.001
Gender		0.738		0.627
female	1		1	
male	1.12(0.48-2.75)		0.81(0.33-1.96)	
Stage		<0.001		<0.001
I	1		1	
II	2.79(0.91-8.61)		2.38(0.72-7.87)	
III	7.77(3.31-18.2)		9.31(3.36-25.78)	
Grade		0.180		0.159
I-II	1		1	
III-IV	1.72(0.78-3.79)		0.51(0.19-1.31)	
Histological type		0.180		
cRCC	1		1	0.355
pRCC	1.34(0.87-2.07)		1.52(0.62-3.65)	

**Internal validation set(N=90)**				
Age		0.06		0.199
<60	1		1	
≥60	3.58(0.95-13.54)		4.25(0.47-38.7)	
17 gene risk score	1.37(1.11-1.69)	<0.001	1.65(1.11-2.48)	0.014
Gender		0.190		0.670
female	1		1	
male	3.97(0.51-31.28)		0.54(0.03-8.97)	
Stage		<0.001		0.005
I	1		1	
II	16.55(1.49-182.7)		48.9(2.63-909.0)	
III	55.86(6.95-448.9)		48.9(4.38-546.0)	
Grade		1.21		0.441
I-II	1		1	
III-IV	2.65(0.77-9.11)		0.491(0.08-2.99)	
Histological type		0.890		
cRCC	1		1	0.209
pRCC	1.05(0.49-2.25)		3.86(0.47-31.72)	

FUSCC: Fudan University Shanghai Cancer Center; ccRCC: clear cell renal cell cancer; pRCC: papillary renal cell cancer.

## References

[1] Bray F, Ferlay J, Soerjomataram I (2018). Global cancer statistics 2018: GLOBOCAN estimates of incidence and mortality worldwide for 36 cancers in 185 countries[J]. CA CANCER J CLIN.

[2] Athar U, Gentile T C (2008). Treatment options for metastatic renal cell carcinoma: a review[J]. CAN J UROL.

[3] Cindolo L, Patard J J, Chiodini P (2005). Comparison of predictive accuracy of four prognostic models for nonmetastatic renal cell carcinoma after nephrectomy: a multicenter European study[J]. CANCER.

[4] Eggener S E, Yossepowitch O, Kundu S (2008). Risk score and metastasectomy independently impact prognosis of patients with recurrent renal cell carcinoma[J]. J UROL.

[5] Ljungberg B, Alamdari F I, Rasmuson T (1999). Follow-up guidelines for nonmetastatic renal cell carcinoma based on the occurrence of metastases after radical nephrectomy[J]. BJU INT.

[6] Richards K A, Abel E J (2016). Surveillance following surgery for nonmetastatic renal cell carcinoma[J]. CURR OPIN UROL.

[7] Kattan M W, Reuter V, Motzer R J (2001). A postoperative prognostic nomogram for renal cell carcinoma[J]. J UROL.

[8] Adamy A, Chong K T, Chade D (2011). Clinical characteristics and outcomes of patients with recurrence 5 years after nephrectomy for localized renal cell carcinoma[J]. J UROL.

[9] Ljungberg B, Bensalah K, Canfield S (2015). EAU guidelines on renal cell carcinoma: 2014 update[J]. EUR UROL.

[10] Giraldo N A, Becht E, Vano Y (2017). Tumor-Infiltrating and Peripheral Blood T-cell Immunophenotypes Predict Early Relapse in Localized Clear Cell Renal Cell Carcinoma[J]. CLIN CANCER RES.

[11] Parker W P, Cheville J C, Frank I (2017). Application of the Stage, Size, Grade, and Necrosis (SSIGN) Score for Clear Cell Renal Cell Carcinoma in Contemporary Patients[J]. EUR UROL.

[12] Slaby O, Redova M, Poprach A (2012). Identification of MicroRNAs associated with early relapse after nephrectomy in renal cell carcinoma patients[J]. GENES CHROMOSOMES CANCER.

[13] Brooks S A, Brannon A R, Parker J S (2014). ClearCode34: A prognostic risk predictor for localized clear cell renal cell carcinoma[J]. EUR UROL.

[14] Rini B, Goddard A, Knezevic D (2015). A 16-gene assay to predict recurrence after surgery in localised renal cell carcinoma: development and validation studies[J]. LANCET ONCOL.

[15] Qu L, Wang Z, Chen Q (2018). Prognostic Value of a Long Non-coding RNA Signature in Localized Clear Cell Renal Cell Carcinoma[J]. EUROPEAN UROLOGY.

[16] Tibshirani R (1997). The lasso method for variable selection in the Cox model[J]. STAT MED.

[17] Pierorazio P M, Johnson M H, Ball M W (2015). Five-year analysis of a multi-institutional prospective clinical trial of delayed intervention and surveillance for small renal masses: the DISSRM registry[J]. EUR UROL.

[18] Zarrabi K, Wu S (2018). Current and Emerging Therapeutic Targets for Metastatic Renal Cell Carcinoma[J]. CURRENT ONCOLOGY REPORTS.

[19] Barata P C, Rini B I (2017). Treatment of renal cell carcinoma: Current status and future directions[J]. CA CANCER J CLIN.

[20] Sun M, Marconi L, Eisen T (2018). Adjuvant Vascular Endothelial Growth Factor-targeted Therapy in Renal Cell Carcinoma: A Systematic Review and Pooled Analysis[J]. EUR UROL.

[21] Brannon A R, Reddy A, Seiler M (2010). Molecular Stratification of Clear Cell Renal Cell Carcinoma by Consensus Clustering Reveals Distinct Subtypes and Survival Patterns[J]. GENES CANCER.

[22] Kosari F, Parker A S, Kube D M (2005). Clear cell renal cell carcinoma: gene expression analyses identify a potential signature for tumor aggressiveness[J]. CLIN CANCER RES.

[23] Chevrier S, Levine J H, Zanotelli V (2017). An Immune Atlas of Clear Cell Renal Cell Carcinoma[J]. CELL.

[24] Negrier S, Perol D, Menetrier-Caux C (2004). Interleukin-6, interleukin-10, and vascular endothelial growth factor in metastatic renal cell carcinoma: prognostic value of interleukin-6-from the Groupe Francais d'Immunotherapie[J]. J CLIN ONCOL.

[25] Hakimi A A, Reznik E, Lee C H (2016). An Integrated Metabolic Atlas of Clear Cell Renal Cell Carcinoma[J]. CANCER CELL.

[26] Morgan T M, Mehra R, Tiemeny P (2018). A Multigene Signature Based on Cell Cycle Proliferation Improves Prediction of Mortality Within 5 Yr of Radical Nephrectomy for Renal Cell Carcinoma[J]. EUR UROL.

[27] Motzer R J, Hutson T E, Hudes G R (2014). Investigation of novel circulating proteins, germ line single-nucleotide polymorphisms, and molecular tumor markers as potential efficacy biomarkers of first-line sunitinib therapy for advanced renal cell carcinoma[J]. CANCER CHEMOTHER PHARMACOL.

[28] Gerlinger M, Rowan A J, Horswell S (2012). Intratumor heterogeneity and branched evolution revealed by multiregion sequencing[J]. N ENGL J MED.

