# Supplementary material for: Development and validation of a robust multigene signature as an aid to predict early relapse in stage I-III clear cell and papillary renal cell cancer
Source: J Cancer. 2020 Jan 1;11(5):997–1007. doi: 10.7150/jca.38274 (PMC6959077; doi:10.7150/jca.38274)
Supplement: Supplementary file 1 — Supplementary tables. [file jcav11p0997s1.pdf]

Supplemental Table 1. The primer sequences for qRT-PCR assay

Gene name	Number	Primer
AFP	F	CTTTGGGCTGCTCGCTATGA
	R	GCATGTTGATTTAACAAGCTGCT
ATP6V0D2	F	TCTCACCTATATGACGTGCAGT
	R	GGTGGCACTTCCCCAGAATTT
COL22A1	F	CCTAGCGTTCGTGTAGAAGGA
	R	CCCATCCGTACATAGGAACTCT
EN2	F	CCGGCGTGGGTCTACTGTA
	R	CCTCTTTGTTCGGGTTCTTCTT
EYA1	F	CACCACAGATTTACCCTTCCAAC
	R	GTACGTGGCATAGGCTGTAGC
HOXA13	F	CTGCCCTATGGCTACTTCGG
	R	CCGGCGGTATCCATGTACT
IGF2BP3	F	TATATCGGAAACCTCAGCGAGA
	R	GGACCGAGTGCTCAACTTCT
IGSF9	F	GAAGCCTGAGGTGGTATCGG
	R	CAGCCACTCGATGACATGC
ITGAD	F	TCGGTGGATCTCGACTCGT
	R	GCAGGAACCCTTTGAGTATGAG
KCNG1	F	ATGACCCTCTTACCGGGAGAC
	R	TGATGCCGCCTACGTTGATG
MT1X	F	TCCTGCAAGAAGAGCTGCTG
	R	TGTCTGACGTCCCTTTGCAG
PGAM2	F	AGAAGCACCCCTACTACAACTC
	R	TCTGGGGAACAATCTCCTCGT
RYR2	F	ACAACAGAAGCTATGCTTGGC
	R	GAGGAGTGTTCGATGACCACC
SLC22A2	F	AGACAGTGTAGGCGCTACGA
	R	CATCGTCACCGAGTTTAACCTG
STRA6	F	CCACAGAGGACTACTCCTATGG
	R	CAGCACAAGGATTGACAGCG
STXBP6	F	TTCTTGGCAACTGGAGGTCAA
	R	TCGATACCATTAACCTGGCGAA
ZIC2	F	GCGCAACTCCACAACCAGTA
	R	TGCCGCATATAGCGGAAAAAG
β-actin	F	CATGTACGTTGCTATCCAGGC
	R	CTCCTTAATGTCACGCACGAT
